# Roy O. Weller (1938–2022); leader of British neuropathology

**DOI:** 10.1111/bpa.13123

**Published:** 2022-10-03

**Authors:** David W. Ellison

**Affiliations:** ^1^ Department of Pathology St. Jude Children's Research Hospital Memphis Tennessee USA

Dear Reader,

Roy Weller, who died on Tuesday 23rd of August at the age of 84 years, was a British neuropathologist with an international reputation for his scientific writings, teaching, and tenure as Editor‐in‐Chief of the journal *Neuropathology and Applied Neurobiology*.

Roy was born in London and grew up there throughout the Second World War, starting medical school at Guy's Hospital in 1956. During his medical studies, he completed an intercalated BSc degree in Anatomy, which was taught by the two editors of Gray's Anatomy. After qualifying and pre‐registration house officer jobs, Roy became a Demonstrator in Anatomy and scientific researcher, investigating the ultrastructural features of demyelinating neuropathies with John Cavanagh and atherosclerosis with Colin Adams [1]. Electron microscopy was then relatively new, and it was typical of Roy to take advantage of the latest research technologies. During this time, Roy also began studying neuropathology with Henry Urich, but this was short‐lived, as a new opportunity beckoned across the Atlantic.

In 1967, having been awarded a fellowship to study at Albert Einstein College in New York, Roy embarked, with his family, for the USA. Guided by Bob Terry and Henryk Wisniewski and again using electron microscopy, Roy studied the progressive effects of acute hydrocephalus on the brain [2]. One consequence of this research was the realization that early use of a shunt in the treatment of patients with hydrocephalus could improve prognosis. By the end of 1968, Roy was back in London, a Lecturer in Pathology at Guy's Hospital. However, he decided to supplement this role with training in neuropathology at the Institute of Psychiatry, thus building a significant clinical and academic skillset that made him the perfect candidate for his definitive appointment.

At the start of 1973, Roy joined the department of Pathology at the University of Southampton School of Medicine, where he would spend the next 30 years as the head of Neuropathology. Both the Wessex Neurological Centre, the regional center for neurosurgery and neurology on the south coast of England, and the University of Southampton School of Medicine were relatively new, and this was a great opportunity for Roy to build the clinical services, research activities, and education for his chosen discipline. From scratch, he built an outstanding clinical neuropathology laboratory, which was well resourced and had extremely high technical standards. It was characteristic of Roy to bring pertinent data into the planning process. When he decided to gauge the nature of the neuro‐oncology service in his new hospital, Roy reviewed the histopathological features of the primary central nervous system (CNS) tumors from the 9 years prior to his arrival! Making further use of the data, Roy, with David Barker and John Garfield, then published the results [3].

Roy was my mentor, and I was privileged to work alongside him in Southampton for just over 10 years, from 1990 to the end of 2000, first as his trainee and then as Consultant colleague. Just after my transition from trainee to Consultant, Roy counseled me on career development. At that time, it was rare for Consultants to move from one position to another. But, despite his devotion to Southampton, Roy saw things a little differently. He was interested in how people navigated their way to professional goals and was keen to emphasize the importance of an evolving career, with new goals and aspirations to keep one motivated and challenged. This advice played a significant role in shaping my future career, and for that I will always be grateful.

In the School of Medicine's academic environment, Roy was a consummate educator, influencing so many across multiple disciplines. Whether the subject was the general principles of pathology or the ultrastructural details of a rare leukodystrophy, Roy had the knowledge and appreciation to teach in a way that both informed and stimulated the interest of his students. He pushed his students to be critical, skeptical, and methodical in their approach to science. Roy saw his teaching as a partnership, an opportunity to engage in Socratic debate. Those who benefited from his knowledge and advice encompassed not only medical students and trainees in pathology, neurology, and neurosurgery at Southampton, but a steady stream of visitors from abroad, including neuropathologists and neuroscientists at different stages of their careers.

Roy's supervision of his trainee's clinical work was both influential and fun. When we met in the evening to review the day's cases, he was as keen to focus on the neurobiology of the disease as the diagnosis on the report. For each case, I would present the clinicopathological background and my histopathological findings. This might have been the most mundane and clear‐cut meningioma or glioblastoma, and Roy could easily have reviewed my diagnosis with a quick “I agree.” But this was not his way. We would study aspects of the tumor's morphology or immunophenotype, looking for interesting features that might lend themselves to a debate about tumor classification or further experimental investigation.

Roy was a great writer. He edited several textbooks in the 1980s, including the volume on CNS pathology in “Systemic Pathology,” which was edited by Bill Symmers. He also contributed to chapters in Greenfield's Neuropathology and early editions of the WHO classification of CNS tumors. Between 1988 and 1998, Roy was Editor‐in‐Chief of Neuropathology and Applied Neurobiology. This was a successful time for the journal, and Roy relished his role, advocating scientific excellence and clear communication and offering advice and encouragement to young authors. Even in retirement, he remained on the editorial board for many years, providing support, ideas, and wisdom. Roy was very attentive to English style and grammar, and I was the beneficiary of advice on transparent writing throughout my time in Southampton.

Research was always important to Roy and, in many ways, bound to his teaching. His studies were about curiosity, not self‐promotion, and Roy was happiest when helping a medical undergraduate or PhD student to test a hypothesis, to conduct a series of experiments, and to report their results concisely. Following on from his early interest in the leptomeninges and their relationship to the brain, his research themes involved studying the lymphatic drainage of the brain, particularly via pathways through the cribriform plate to the cervical lymph nodes, and the accumulation of amyloid‐ß in artery walls in the brain of patients with cerebral amyloid angiopathy or Alzheimer disease [4]. Early in my training, he pushed me to present our work on p53 in astrocytic tumors at international meetings [5]. Roy was an enthusiastic supporter of conferences and seemed to know everyone! This outgoing approach was typical of Roy; he loved to learn about the latest research findings and to spend time debating their significance. As such, he will be remembered by many friends in the neuroscience community, not just neuropathologists.

Roy retired from his position in Southampton in 2003, at a time when there was a mandatory retirement age from the National Health Service and British universities. But he wasn't ready. Roy felt that he had more to give and attended the Medical School on a voluntary basis, teaching and supporting research. Characteristically, this was highly successful. He continued to advise on studies related to his research interests, especially influential work from Roxana Carare's group [6], and appeared as a co‐author on her papers as recently as last year. In retirement, Roy kept up his interests in music and also signed up to be a guide at Winchester Cathedral. Winchester was home to Roy and his family during his tenure at Southampton, and its ancient cathedral is a wonderful place to visit. Just a few years ago, I visited Roy and his wife, Francine, in Winchester and was taken on a special tour of the cathedral that included a narrow walkway under the main roof, but above the ceiling of the nave, and a visit to the roof itself. The views were spectacular! The experience reminded me of Roy's talents: he had carefully plotted a detailed path to a goal, to be rewarded by a broad vision—whether the analysis of a specific facet of neuropathology or the wonders of his hometown.

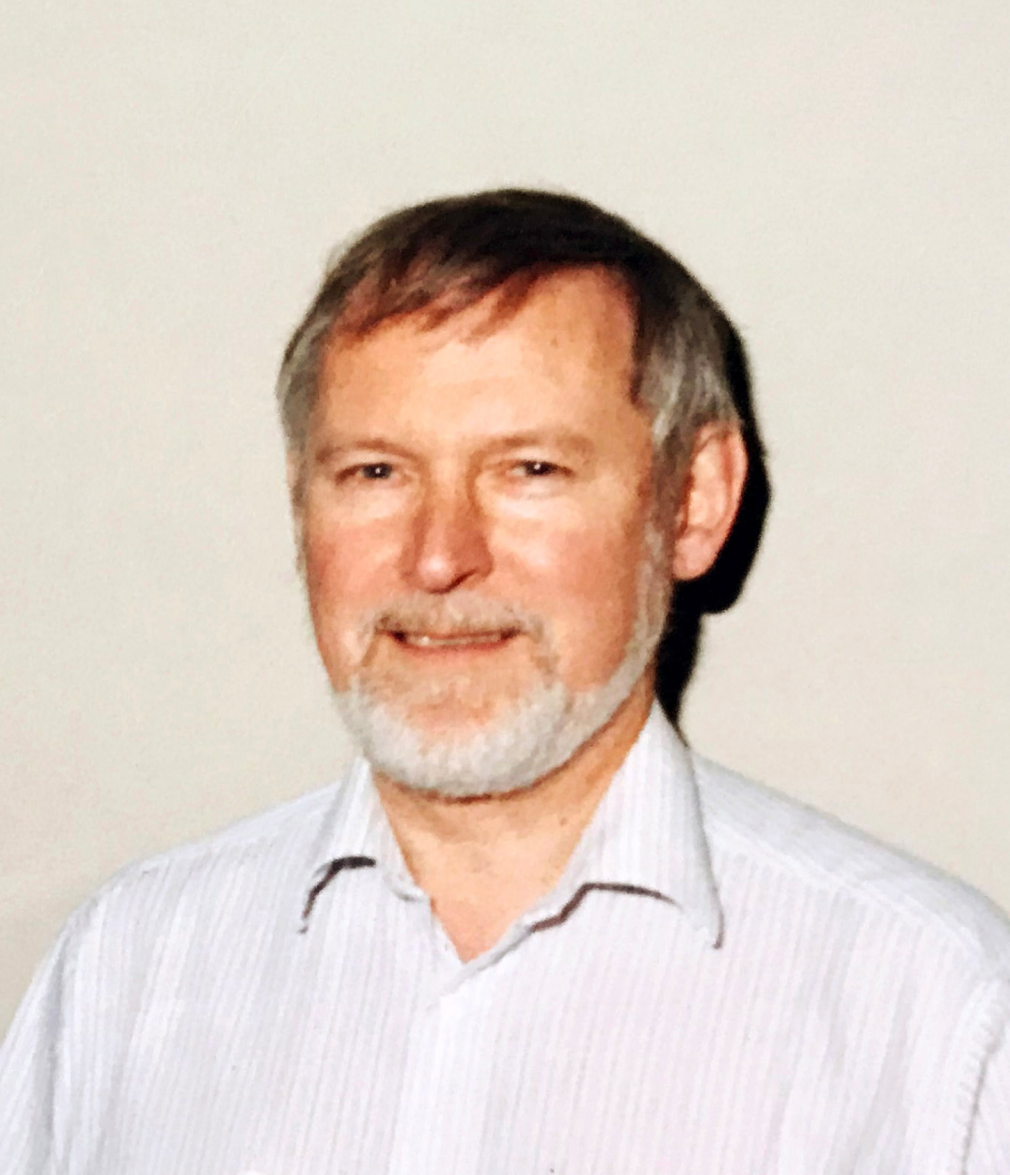

## CONFLICT OF INTEREST

The author declares there is no potential conflict of interest.

## Data Availability

Data sharing is not applicable to this article as no new data were created or analyzed in this study.
